# Proteomics Analysis of the Non-Muscle Myosin Heavy Chain IIa-Enriched Actin-Myosin Complex Reveals Multiple Functions within the Podocyte

**DOI:** 10.1371/journal.pone.0100660

**Published:** 2014-06-20

**Authors:** Thomas Hays, Avi Ma’ayan, Neil R. Clark, Christopher M. Tan, Avelino Teixeira, Angela Teixeira, Jae W. Choi, Nora Burdis, Sung Yun Jung, Amol O. Bajaj, Bert W. O’Malley, John C. He, Deborah P. Hyink, Paul E. Klotman

**Affiliations:** 1 Department of Medicine, Icahn School of Medicine at Mount Sinai, New York, New York, United States of Americ; 2 Department of Pharmacology and Systems Therapeutics, Icahn School of Medicine at Mount Sinai, New York, New York, United States of America; 3 Department of Medicine, Baylor College of Medicine, Houston, Texas, United States of America; 4 Department of Molecular and Cellular Biology, Baylor College of Medicine, Houston, Texas, United States of America; University of Houston, United States of America

## Abstract

*MYH9* encodes non-muscle myosin heavy chain IIA (NMMHCIIA), the predominant force-generating ATPase in non-muscle cells. Several lines of evidence implicate a role for *MYH9* in podocytopathies. However, NMMHCIIA‘s function in podocytes remains unknown. To better understand this function, we performed immuno-precipitation followed by mass-spectrometry proteomics to identify proteins interacting with the NMMHCIIA-enriched actin-myosin complexes. Computational analyses revealed that these proteins belong to functional networks including regulators of cytoskeletal organization, metabolism and networks regulated by the HIV-1 gene *nef*. We further characterized the subcellular localization of NMMHCIIA within podocytes *in vivo*, and found it to be present within the podocyte major foot processes. Finally, we tested the effect of loss of *MYH9* expression in podocytes *in vitro*, and found that it was necessary for cytoskeletal organization. Our results provide the first survey of NMMHCIIA-enriched actin-myosin-interacting proteins within the podocyte, demonstrating the important role of NMMHCIIA in organizing the elaborate cytoskeleton structure of podocytes. Our characterization of NMMHCIIA’s functions goes beyond the podocyte, providing important insights into its general molecular role.

## Introduction

Myosin II is the predominant mammalian force-generating ATPase. It is a heterohexamer composed of two heavy chains of NMMHCIIA, two essential light chains and two regulatory light chains. The N-terminus of NMMHCIIA is a flexible globular motor domain, which binds and translocates actin filaments. Its C-terminal coiled-coil domain mediates protein-protein interactions (PPIs). NMMHCIIA mediates cytoskeletal organization, cell migration, cell contractility and vesicle trafficking [1], [2]. Rare mutations in *MYH9* cause the syndrome known as MYH9-related disorders (MYH9RD), which includes a variably penetrant podocytopathy characterized by irregular thickening of the glomerular basement membrane (GBM), proteinuria and renal failure [3]–[5].

Single nucleotide polymorphisms (SNPs) in *MYH9* were found to predict susceptibility to HIV-associated nephropathy (HIVAN), focal segmental glomerulosclerosis (FSGS) and endstage renal disease [6], [7]. These SNPs were determined to be sentinels for risk alleles of the neighboring gene, *APOL1,* which demonstrated a stronger link to kidney disease [8]. A mechanism by which *APOL1* mediates disease is yet unknown. Furthermore, several recent investigations have implicated a role for *MYH9* in mediating kidney disease independent of *APOL1.* Genetic analyses linked variants in *MYH9* to chronic kidney disease in populations lacking the *APOL1* risk alleles [9]. *MYH9* SNPs independently associate with a risk of sickle cell nephropathy [10]. Additionally, glomerular expression of *MYH9* is down-regulated by HIV-1 in HIVAN [11]. Finally, it has been shown that ablation of *MYH9* in podocytes predisposes mice to adriamycin-induced nephropathy [12]. Given the unclear genetic picture, elucidating the contribution of *APOL1* and *MYH9* at this locus requires a clearer understanding of the molecular functions of these genes within the podocyte. Given the body of evidence linking *MYH9* to renal processes and podocytopathy in particular, we chose to investigate the protein-protein interactions (PPIs) of the NMMHCIIA-enriched fraction of actin-myosin-interacting proteins within podocytes.

Podocytes have an elaborate cytoskeleton and interdigitating foot processes overlying the glomerular basement membrane (GBM) [13]. Their function in renal filtration is facilitated by extracellular slit diaphragm proteins, which depend on the underlying actin cytoskeleton and a network of linking proteins [14]. We hypothesized that podocyte-expressed proteins form functional networks in which NMMHCIIA plays a necessary role. To better understand NMMHCIIA’s functional role, and to catalog and analyze the function of proteins within the NMMHCIIA-enriched actin-myosin complexes of podocytes, we generated NMMHCIIA cross-linked, immuno-precipitated samples using cultured podocytes. We then performed mass-spectrometry (IP-MS) proteomics, followed by network analysis to determine which protein complexes NMMHCIIA might participate in, and to explore the functions of these complexes.

The network analysis that was performed seeded the identified proteins from the IP-MS studies within the known PPI network from the literature. This enables the formation of a sub-network made of putative NMMHCIIA interacting proteins. Community structure analysis of this sub-network was then applied to identify clusters of interacting proteins. When a set of connected proteins contains a statistically significantly higher degree of connectivity, it is assumed that this represents a meaningful functional complex. Furthermore, community structure analysis allows the detection of the enrichment of gene ontology (GO) terms [40] or other functional categories associated with gene sets [57] to predict complex function. Analyzing GO or other functional terms that are enriched in these clusters to a statistically significant degree can indicate the functional significance of the protein complexes.

Our computational analysis revealed pathways which regulate cytoskeletal organization, metabolism, and networks regulated by the HIV-1 gene *nef.* To gain further insight into the *in vivo* localization of NMMHCIIA within podocytes, we analyzed its localization by immunofluorescence, and found it to be within glomerular capillary tufts. Given these findings, we tested the effect of *MYH9* knockdown on podocyte cytoskeletal reorganization, and found that decreased expression of *MYH9* resulted in loss of actin-myosin stress fibers, a typical feature of differentiated podocytes. Our results provide important insights into the molecular functions of NMMHCIIA in the podocyte, specifically the involvement of NMMHCIIA with the Rho cytoskeletal regulating pathway.

## Materials and Methods

### Screening for NMMHCIIA Interacting Proteins

Murine conditionally immortalized podocytes, which proliferate at growth permissive (GP) conditions, and express differentiation characteristics at growth restrictive (GR) conditions, were maintained as previously described [15], [16]. Health of podocytes was monitored morphologically as previously described [16]. Following one week of growth at GR conditions, protein was extracted from two T75s containing approximately 1×10^6^ cells using T-Per protein extraction reagent supplemented with 1×protease inhibitor cocktail (Pierce, Thermo Scientific, Rockford, IL, Catalog #78510 and 78430). Total protein lysate was then cross-linked in paraformaldehye. Columns made using rabbit polyclonal antibody directed against the C-terminal dodecapeptide of NMMHCIIA (Sigma, Catalog #M 8064) were made to immunoprecipitate NMMHCIIA and crosslinked proteins.

Total protein was denatured in 8 M urea/100 mM NH_4_HCO_3 _pH 8, reduced with DTT, acetylated with acetamide and digested with trypsin (16 hours). Tryptic peptides were analyzed by LC/MS^5^ on an LTQ linear ion trap mass spectrometer (ThermoFinnigan, San Jose, CA). Mass spectra of the tryptic peptides derived from tandem mass spectrometry were searched against theoretical spectra derived from the human nonredundant database (NCBI, Bethesda, MD) using the SEQUEST algorithm function of the Bioworks (ThermoFinnigan) mass spectra data analysis software and manually validated. Fragmentation spectra were used from the 25 strongest ions. The precursor mass tolerance was confined within 20 ppm with fragment mass tolerance of 0.5 dalton and a maximum of two missed cleavage allowed. Assigned peptides were filtered with 5% false discover rate (FDR) and subject to manual verifications. In order to increase the specificity of this screen, and to detect interactions in a model of human podocytes, we repeated this approach using human conditionally immortalized podocytes, which were cultured at GP and GR conditions as previously described [15]–[17].

### Computational Analyses

Proteins identified by IP-MS were connected using known protein-protein interactions (PPIs) from publicly available sources including the following databases and publications: BioGrid [18], HPRD [19], InnateDB [20], IntAct [21], KEGG [22], KEA [23], MINT [24], MIPS [25], DIP [26], BIND [27], BioCarta, PDZBase [28] and PPID [29]–[33]. For the pathway enrichment analysis we used Lists2Networks [34] to compute enrichment for BioCarta pathways, Gene Ontology [40], WikiPathways [35], as well as several other gene set libraries we developed [57].

Community structure within the NMMHCIIA PPI subnetwork was characterized by connecting proteins identified in our screen with known PPIs from the literature [36]. The algorithm begins with each node belonging to its own community. Then the algorithm iteratively merges communities and this leads to an increase in the community modularity *Q*. The community modularity as nodes partitioned into *k* distinct communities with sum:
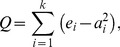
(1)where 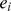 is the fraction of all edges which connect nodes belonging to the 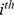
community, and 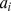 is the fraction of all edges that connect one node from the 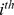 community with a node outside of that community [36]. The idea behind this clustering approach is the identification of areas within the protein interaction network that are densely connected. We wish to identify clusters of proteins that are highly connected to each other while loosely connected to other clusters. These complexes can then be evaluated for their shared functional purpose using enrichment analysis. The algorithm is a classical algorithm in the network analysis literature [36].

Clusters were then analyzed for representation of known protein complexes in the Comprehensive Resource of Mammalian Protein Complexes (CORUM) [37] and for enrichment of GO terms and pathways with Lists2Networks [34].

To identify relationships between the identified proteins we used the program Genes2FANs [38] to create a subnetwork where the interactions are functional rather than physical. Genes2FANs uses 14 different gene-set libraries to identify functional relationships between genes/proteins based on co-occurrence in gene sets within gene set libraries. For example, genes can be connected if they co-express, share GO terms, are targets of the same microRNAs, display the same mouse phenotypes when knockout, disease genes sharing the same diseases, or have shared structural domains.

To characterize the density of the human and murine networks of proteins, random samples of proteins from PPIs were repeatedly generated, and the number of known interactions was counted to estimate the probability distribution function (PDF). The number of known interactions between proteins identified in our screen was compared to the background PDF for a random sample of proteins. To determine whether the connectivity in the networks was the result of a small subset of proteins participating in promiscuously large numbers of interactions, we estimated the degree distribution of the identified proteins, and compared these to the degree distribution of the PPI network as a whole.

Enrichment analysis of the two lists was performed with Lists2Networks [34] and Network2Canvas [56] to compute enrichment for WikiPathways [35], BioCarta pathways, Reactome [39], PPI hubs, KEA [23], GO [40], VirusMINT [41], and structural domains from PFAM [42] and InterPro [43]. These tools use the Fisher exact test and the gene-set libraries to rank terms based on overlap of the identified protein subset with gene-sets selected from each gene-set library. To visualize the results on a square grid display we used Network2Canvas [56]. To characterize networks of proteins known to have roles mediating disease phenotypes, we searched the OMIM database for previously described interactions containing the identified human proteins [44].

### Histology

To localize NMMHCIIA expression, nephrectomy specimens were obtained from patients undergoing partial or whole nephrectomies for renal cell carcinoma (RCC]. Cortical tissue unaffected by RCC was fixed in 10% neutral buffered formalin, and stained for NMMHCIIA by immunofluorescence and counterstained with phalloidin and 4′,6′-diamidino-2-phenylindole (DAPI) as previously described [11]. Wide field immunofluorescent images of glomerular NMMHCIIA expression were produced by deconvolution using AutoQuant X2. Work with human tissue was approved by the Institutional Review Board of Icahn School of Medicine at Mount Sinai, which waived the need for patient consent (protocol: 98-953 0001 10ME].

### *MYH9* Knockdown

shRNA constructs against *MYH9* were utilized (Origene, Rockville, MD, Catalog #TF311309, constructs 1–4]. To determine, which construct achieved optimal knockdown of *MYH9* expression, 3 µg of each vector, and a scrambled shRNA construct, was transfected into freshly passaged 293T cells growing at 75% confluencey in each well of a 6-well plastic plate using Genejuice transfection reagent (Novagen, Merck KGaA, Darmstadt, Germany). Four days following transfection, total protein was isolated in T-Per protein extraction reagent with 1×protease inhibitor cocktail (Pierce, Thermo Scientific, Rockford, IL, Catalog #78510 and 78430). Total protein was quantified by BCA assay, and 10 µg was run by SDS-PAGE on Novex tris-glycine 4–20% gels under denaturing conditions (Invitrogen, Catalog #EC6025). Protein was transferred to PVDF membrane by iBlot transfer (Invitrogen). Membranes were blocked for 30 minutes in PBS/0.1% Tween-20/5% powdered non-fat milk. Membranes were blotted with rabbit anti-NMMHCIIA antibody overnight at 1∶1000 dilution at 4°C (Sigma, Catalog #M 8064). Following overnight primary incubation and three washes in PBS/0.1% Tween-20, membranes were incubated in 1∶10,000 HRP-conjugated donkey anti-rabbit secondary antibody (Jackson ImmunoResearch Laboratories, Catalog #711-036-152) for two hours at room temperature. Bands were detected using Lumi-Light Western blotting substrate (Roche, Catalog #12015200001). Membranes were stripped using Restore Plus stripping buffer (Pierce) then blotted as before using a 1∶5000 diluted mouse anti-GAPDH primary antibody (Millipore, Catalog #Mab 374), and a 1∶200 diluted HRP-conjugated anti-mouse secondary antibody (Zymax, Catalog #81-6520).

Human conditionally immortalized podocytes grown in GP conditions were transfected with the shRNA-2 construct against *MYH9*, and with the scrambled shRNA-NC control. Transfections were performed with the Neon Transfection system (Invitrogen, Carlsbad, CA). 8×10^6^ cells per transfection were suspended with 10 µg of each DNA encoding shRNA against MYH9 or 10 µg of scrambled shRNA plasmid in buffer R2 to a final volume of 100 µL, which was then transfected using a 1400 V pulse for 20 ms. Cells were plated in collagenized 6-well plates or glass slides at a density of 3×10^4^ cells per cm^2^ overnight in GP conditions. Cells were shifted to 37°C growth restrictive (GR) conditions for 7 days. Cells were stained for actin as previously described [45]. Total RNA was analyzed by real-time PCR for expression of *18S* and *MYH9* as above. Cells were stained using DAPI and Alexa Fluor 555 Phalloidin as previously described [45].

## Results

### Screen for NMMHCIIA-interacting Proteins

**Figure 1:**
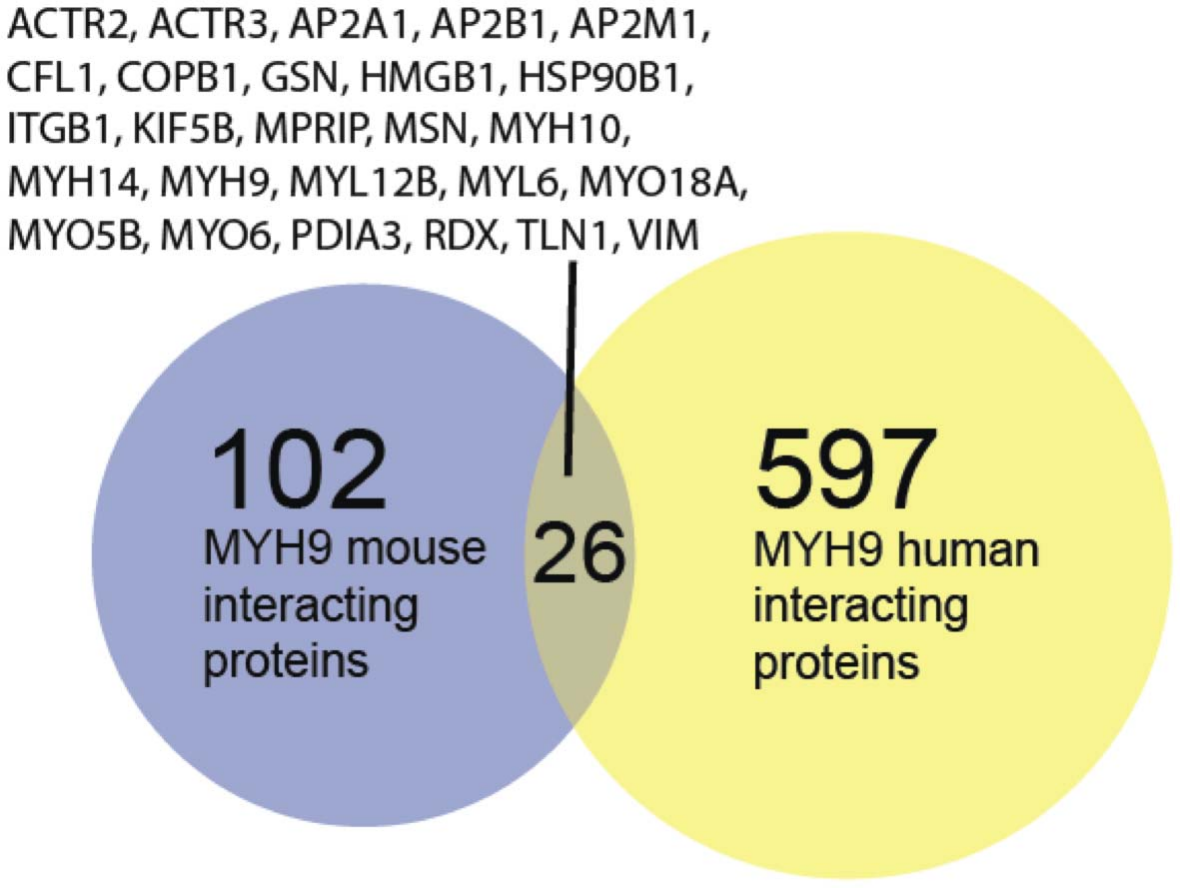
Venn diagram of the identified proteins by the IP-MS studies.

**Figure 2:**
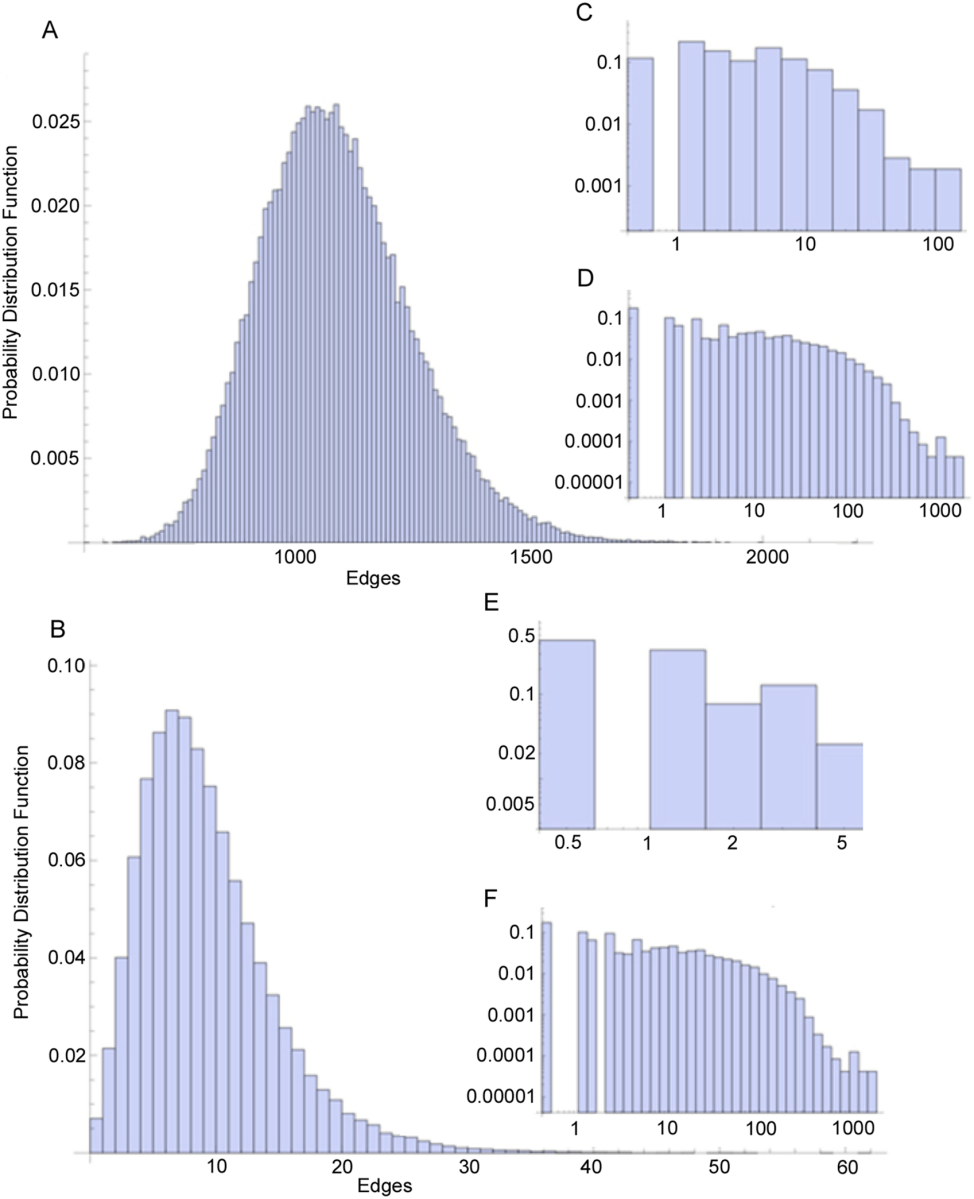
Statistical characterization of MYH9-interacting proteins. We compiled a database of known human protein-protein interactions (PPI) and used this as a basis from which to evaluate the density of interactions between the human and murine proteins identified by IP-MS. There are 1269 human proteins identified by the IP-MS and in the database there are 5605 interactions between them. In order to ascertain if this number of interactions is larger than what might be expected by chance we selected (n = 10^5^) random sets of 1269 proteins from the database and counted the number of interactions between them, the probability density is shown in (A). Obviously the observed number of 5505 proteins occupies an extreme (large) position in this distribution, having a p value p<10^−5^. In order to determine if an overrepresentation of “hub” proteins is responsible for this extreme density we compared the degree distribution of the subgraph of the PPI network induced by the identified human proteins (C) to the degree distribution of the whole PPI network (D); the similarity between the two distributions indicates that there is no overrepresentation of hub proteins. This analysis was repeated for the (N = 128) identified murine proteins where the distribution of the number of interactions is shown in (B) and the actual number of interactions is 50 (p<10^−5^), and the corresponding degree distributions of the induced subgraph and whole PPI network are shown in (E) and (F) respectively. These figures reveal similar results as for the human proteins.

Our analysis identified 128 proteins in murine podocytes, and 623 (623 high confidence out of 1269 total) proteins in human podocytes as potential proteins which interact with the NMMHCIIA-enriched actin-myosin complex (Tables S1 and S2). 26 proteins appeared in both screens (Figure 1), which is much more than expected by chance (p<10^−5^, Fisher exact test). We then used known PPIs from the literature to examine if there is independent evidence that the proteins we experimentally identified physically interact with each other. For this we combined and processed available online resources which listed experimentally verified PPIs from the literature and extracted all direct interactions between the proteins we identified within the NMMHCIIA-enriched actin-myosin complex. Comparing the density of PPIs among the identified human proteins with known PPIs between randomly selected proteins revealed that the identified proteins included significantly more PPIs than expected by chance (Figure 2; p<10^−5^, Fisher exact test). The compiled database of known human protein-protein interactions (PPI) was used as a basis from which to evaluate the density of interactions between the human and murine proteins identified by IP-MS. There are 1269 total human proteins identified by the IP-MS and in the literature-based PPIs database there are 5605 known interactions between them. In order to ascertain if this number of interactions is larger than what might be expected by chance we selected (n = 10^5^) random sets of 1269 proteins from the database and counted the number of interactions detected between them. The probability density for such analysis is shown in Figure 2A. The observed number of 5505 proteins occupies an extreme position in this distribution, having a p value p<10^−5^. In order to determine if an overrepresentation of “hub” proteins is responsible for this extreme density, we compared the degree distribution of the sub-network of the PPI network induced by the identified human proteins (Figure 2C) to the degree distribution of the whole PPI network (Figure 2D); the similarity between the two distributions indicates that there is no overrepresentation of hub proteins. This analysis was repeated for the (N = 128) identified murine proteins where the distribution of the number of interactions is shown in (Figure 2B) and the actual number of interactions is 50 (p<10^−5^), and the corresponding degree distributions of the induced sub-network and whole PPI network are shown in (Figure 2E) and (Figure 2F) respectively. These figures reveal similar results for the human and mouse proteins.

Furthermore, these proteins included genes with defined roles in deafness, anemia, myopathy and leukemia as defined in the Online Mendelian Inheritance in Man (OMIM) database (Table 1).

**Table 1:** OMIM disease networks identified in MYH9 interactions.

OMIM Disease Term	Gene
Deafness	DFNA5
	MYH14
	ACTG1
	MYH9
	RDX
Anemia	TPI1
	HK1
	GSS
	GPI
Myopathy	TPM2
	TPM3
	FLNC
Leukemia	LPP
	NUMA1
	PML

Genes identified in the human MYH9 proteomics that are also listed in OMIM as genes that when mutated can cause human diseases. There are many more genes that overlap with entries in OMIM. Only relevant diseases for the discussion are shown.

**Figure 3:**
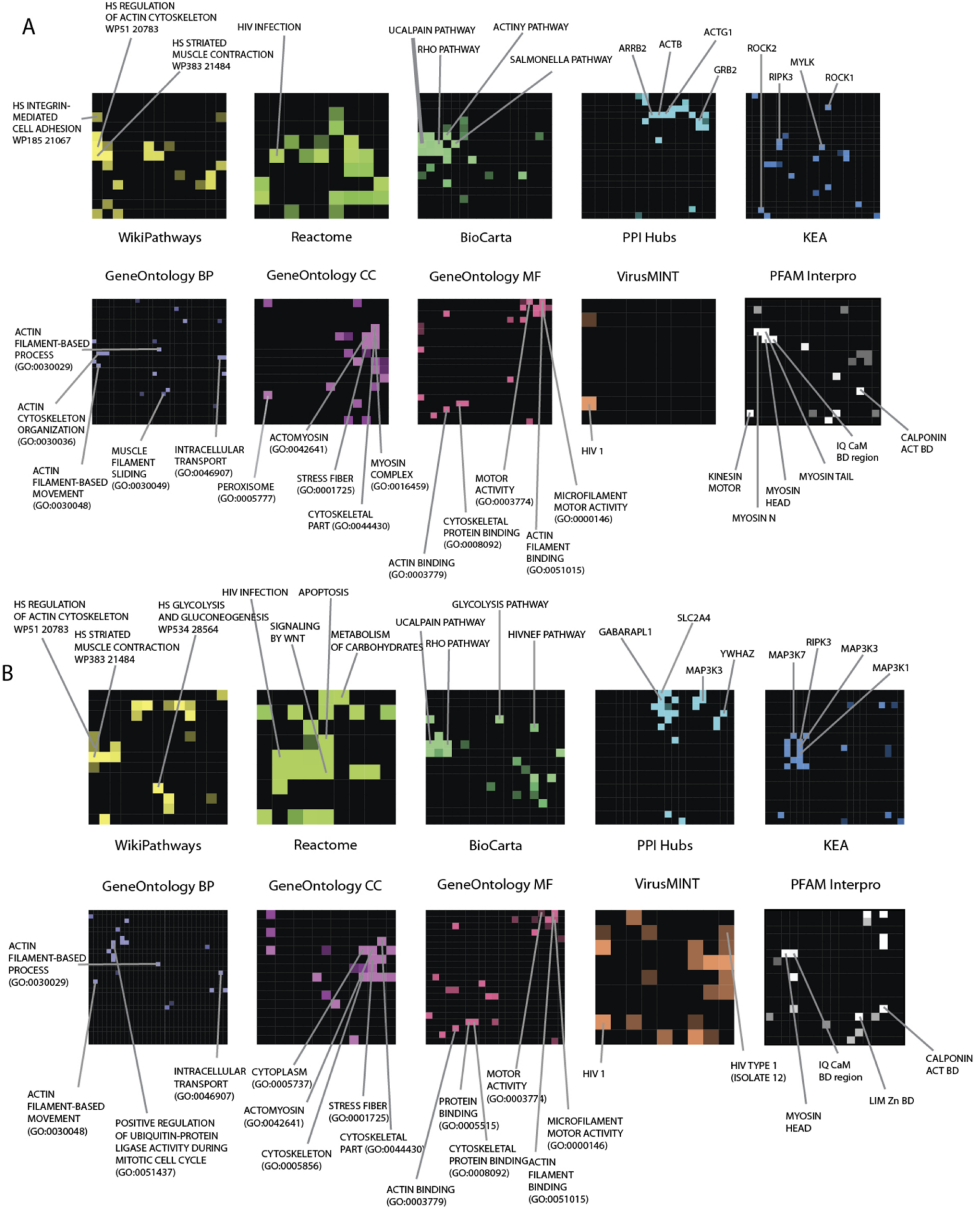
Enrichment analysis with Network2Canvas of identified proteins interacting with MYH9. Eight different gene set libraries: WikiPathways, Reactome, BioCarta pathways, PPI hubs, Kinase Enrichment Analysis (KEA), GO biological process (BP), GO cellular component, GO molecular function, VirusMINT, and protein domains from PFAM and InterPro. On each grid all the terms from the gene set libraries are arranged based on their gene content similarity. The highlighted terms are enriched terms where the brighter colors denote higher significance. Some relevant terms are annotated. A) Analysis of mouse MYH9 interacting proteins; B) Analysis of human MYH9 interacting proteins.

Pathway enrichment analysis using gene-set libraries in human and mouse indicated identified proteins were enriched for gene sets in multiple functional pathways (Figure 3, Tables S3 and S4; p<0.05, Fisher exact test). The enrichment results recapitulate previously described functions for NMMHCIIA, such as regulation of the actin cytoskeleton, vesicle and organelle localization, and additionally identified metabolic pathways such as glycolysis. There are 26 proteins that are shared among the human and mouse proteomics analyses. These proteins are more likely to be important and consistent components of the NMMHCIIA interaction complex. We performed enrichment analysis on these proteins (Tables S3 and S4). These proteins are related to cytoskeleton regulation, intracellular transport, and are enriched for Rho related signaling components (p<0.05, Fisher exact test).

**Figure 4:**
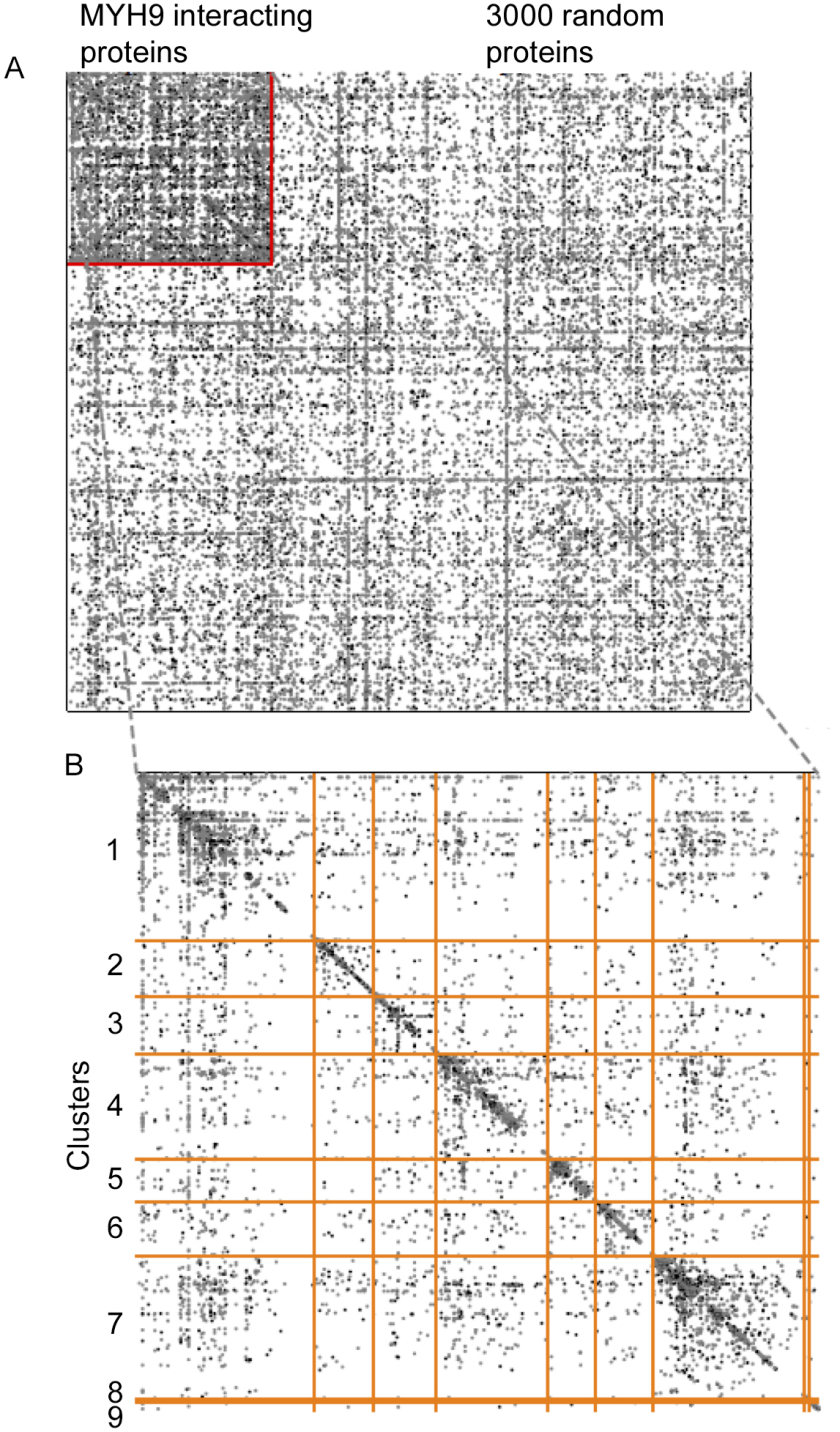
The adjacency matrix of the network of known interactions between the set of MYH9 interacting human proteins, and 3000 random proteins for comparison. The 623 proteins identified as interacting with MYH9 (in the upper left) along with random set of 3000 random human proteins are plotted along both the *x* and *y* axes. Previously described interactions amongst these proteins are depicted as a node at the *x*, *y* intersection of a given pair of proteins (A). The increased density of interactions (visually evident as a higher density of nodes in the upper left) within the set of MYH9-interacting proteins indicates that these proteins also belong to previously described complexes. A close-up view is provided which also displays the community structure, with discrete clusters boundaries indicated with orange lines (B). This community structure indicated that nine distinct clusters exist, representing nine distinct groups of proteins with multiple previously described interactions.

Community structure analysis of the human proteins demonstrated that these proteins belonged to significantly more PPIs that comparing to the average for the entire literature-based PPI network. This is visualized in figure 4 where the proteins within the MYH9 complex are compared to interactions within a random set of 3000 human proteins (Figure 4; p<10^−5^, Fisher exact test). Analysis of connectivity density of PPIs within the set of identified proteins showed that these belonged to nine clusters (Figure 4B). These clusters revealed known CORUM protein complexes and enrichment of GO terms (Table S5). Clusters 1 and 2 were enriched in complexes known to regulate actin cytoskeleton and motility (top GO terms: “cytoskeletal organization” and “cell motion;” p<10^−6^, Fisher exact test). Clusters 3 and 8 contained complexes with known roles in amino acid and nucleotide metabolism and translation (top GO terms: “translational initiation” and “peptide cross-linking;” p<10^−5^ and 0.01 respectively). Cluster 4 contained complexes known to regulate mitosis (top GO term: “mitotic spindle organization;” p<10^−4^). Cluster 5 consisted of complexes involved in ubiquitination and protein turnover (top GO term: “negative regulation of ubiquitin-protein ligase activity during mitotic cell cycle;” p<10^−30^). Clusters 6 and 9 contained complexes involved in vesicle transport (top GO terms: “retrograde vesicle-mediated transport, Golgi to ER” and “intracellular transport;” p<10^−13^ and 10^−6^ respectively). And cluster 7 consisted of complexes that mediate transcription (top GO term: “RNA splicing;” p<10^−24^). These results highlight the postulated notion that proteins within the NMMHCIIA-enriched portion of actin-myosin interactors serve various functions within the cell including cytoskeleton rearrangement, vesicle transport, mitosis, transcription and translation.

### Histologic Analysis of NMMHCIIA Expression

Given the finding of roles for identified proteins in cytoplasmic processes, we sought to analyze the spatial expression of NMMHCIIA in human glomeruli. Consistent with NMMHCIIA’s postulated role in podocyte function, we sought to detect if NMMHCIIA is expressed within podocytes, specifically within the cytoplasmic space of podocyte major processes.

**Figure 5:**
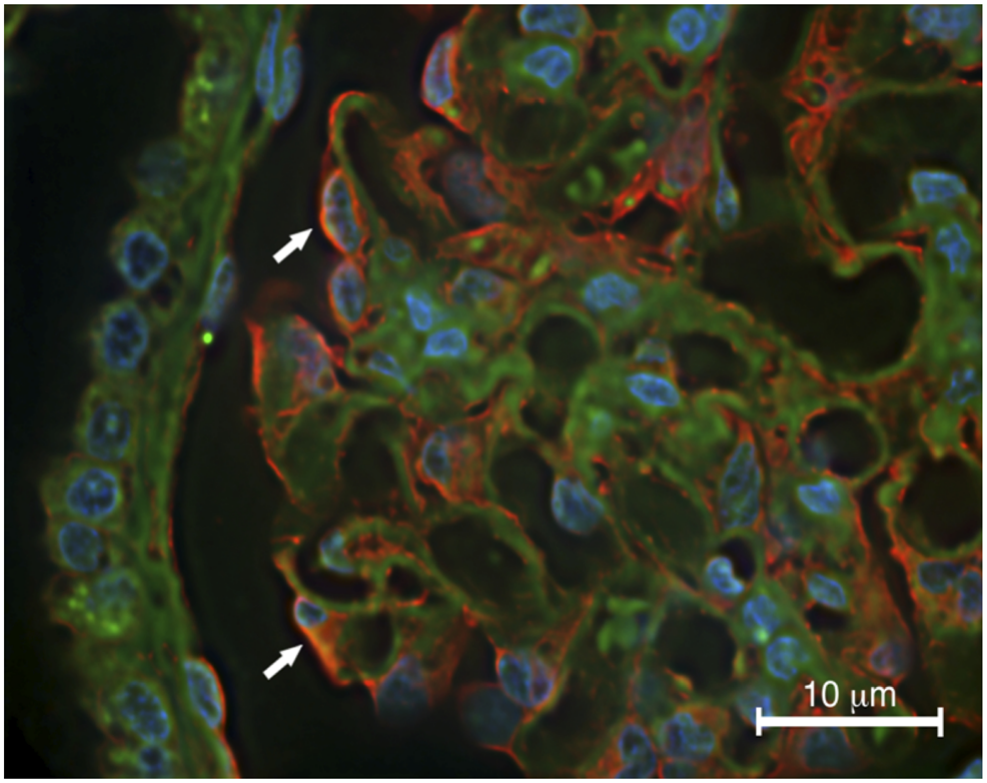
MYH9 is expressed within podocyte processes. Human renal tissue was stained by immunofluorescence (red) as well as phalloidin (green) and DAPI counterstains. MYH9 expression was detected in podocytes (arrows) as well as mesangial cells and parietal epithelial cells. MYH9 appeared to be expressed within the major processes of podocytes overlaying glomerular capillary tufts.

NMMHCIIA has previously been shown to be expressed in healthy human glomeruli, within podocytes specifically [11]. However, it’s subcellular localization has not been described. Higher resolution images by Johnstone, *et al.* demonstrated NMMHCIIA expression within the cytoplasm of murine podocytes [12]. Combining wide field immunofluorescence microscopy with image deconvolution allowed higher resolution images to be obtained. These demonstrated NMMHCIIA expression in podocytes, as previously described, however they further revealed NMMHCIIA expression in podocyte cytoplasm, podocyte major processes overlying glomerular capillary tufts, and in glomerular mesangial cells and parietal epithelial cells (Figure 5). NMMHCIIA expression was excluded from the nuclei of cells in the tissue analyzed.

### Effect of Loss of *MYH9* Expression on Cytoskeletal Organization

The findings that identified proteins are enriched for cytoskeletal regulators combined with the finding that NMMHCIIA is highly expressed in podocyte major processes led us to test whether NMMHCIIA expression was necessary for the formation of the podocyte cytoskeleton *in vitro.* Additionally, a cardinal feature of podocytopathy in the setting of HIVAN is a transition from Rho to Rac-mediated arrangement of the actin cytoskeleton [45], [46]. Given that pathway enrichment analysis detected pathways involving Rho-mediated regulation of the actin cytoskeleton, we sought to analyze the appearance of podocytes in the absence of NMMHCIIA expression, hypothesizing that loss of NMMHCIIA function would lead to a loss of Rho-mediated normal podocyte cytoskeleton.

**Figure 6:**
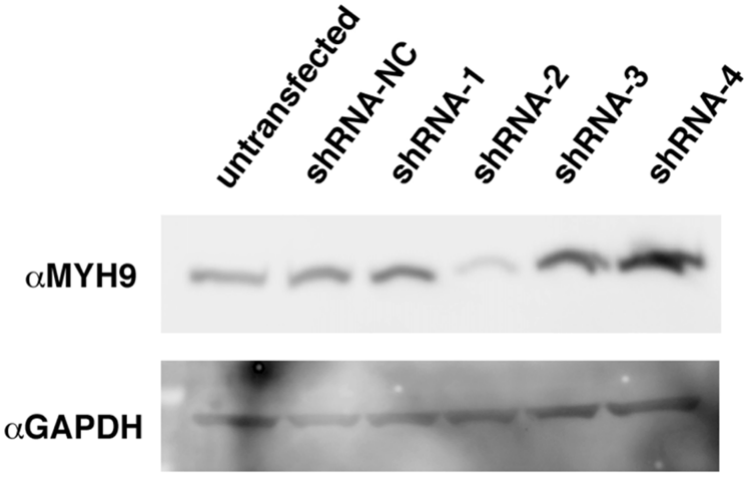
Validation of shRNA constructs against *MYH9.* MYH9 expression was assayed in total protein collected from 293T cells, four days following transfection with shRNA constructs targeting *MYH9* mRNA (shRNAs 1–4). A construct bearing a random shRNA served as negative control (shRNA-NC). Construct 2 achieved a dramatic reduction in expression, while the other constructs did not reduce protein levels.

**Figure 7:**
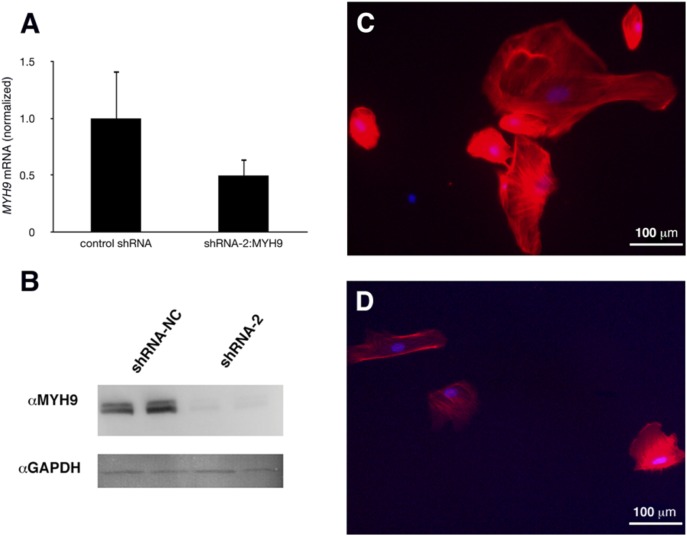
Effect of knockdown of *MYH9* expression on podocytes. Human conditionally immortalized podocytes were transfected with shRNA-2 to reduce *MYH9* expression or shRNA-NC as negative control. Following transfection, podocytes were grown at GR conditions to allow differentiation. Analysis by Real Time-PCR (A) and western blot (B) confirmed knockdown of expression. Podocytes were stained with phalloidin (red) to visualize the actin cytoskeleton and DAPI counterstain. Control podocytes exhibited large cell bodies, with stress fibers typical of differentiated cells (C). Podocytes with reduced *MYH9* expression demonstrated smaller cell size with rarefied actin cytoskeleton, and lacking stress fibers typical of differentiation (D).

We first validated shRNA constructs targeting *MYH9* and found that construct, shRNA-2, produced a robust knockdown of protein expression (Figure 6). Following shRNA-mediated knockdown of *MYH9* mRNA and protein in human conditionally immortalized podocytes (Figures 7A and 7B), the cells exhibited a decrease in cell size, rarefied actin content, and loss of actin stress fiber organization (Figure 7C). Podocytes, transfected with a control scrambled shRNA exhibited actin stress fiber organization typical of Rho-mediated signaling, consistent with the healthy podocyte cytoskeleton (Figure 7C).

## Discussion

Here we highlight two open questions in the field of renal medicine: how does the African derived chromosome 22 allele contribute to renal disease, and why is NMMHCIIA necessary to maintain podocyte function? In reference to the first open question, genetic discoveries have clearly established that variant alleles of *APOL1* were positively selected in Africa because they conferred resistance to trypanosomiasis [8]. Furthermore, genetic investigations have clearly established that the genomic region containing *APOL1* confers susceptibility to kidney disease [6]–[8]. However genetic and genomic investigations have failed to uncover the molecular explanation of the pathogenic mechanism for renal disease.

With regards to the second open question, several lines of evidence indicate that NMMHCIIA is a key regulator of podocyte function. Rare *MYH9* mutations cause a podocytopathy. This pathogenesis likely results from a loss of function, as the most severe renal phenotype is caused by a dominant negative mutation [5], [47]. Loss of expression of *Myh9* in podocytes predisposed mice to adriamycin-induced glomerulopathy [12]. The role that common SNPs in *MYH9* play in glomerular diseases is less clear. A parsimonious explanation remains that *MYH9* SNPs are sentinel markers for *APOL1* alleles. However, genome-wide analyses have found associations between *MYH9* SNPs and glomerular disease independent of *APOL1* [9], [10].

These two open lines of investigation converge at the question of what is the function of NMMHCIIA in podocytes? Proteomic screening of NMMHCIIA-enriched proteins combined with bioinformatics provides a useful means to investigate NMMHCIIA’s molecular function. Specifically, community structure analysis has the power to detect which proteins are found in the NMMHCIIA-enriched fraction of protein complexes within the podocyte. By analyzing the enrichment of GO terms associated with these genes, functional roles of identified proteins can be determined. By using this approach, we found that *MYH9* interacts with protein networks including those of Rho, which mediates podocyte cytoskeletal structure and function, networks regulated by the HIV-1 gene *nef* (a key mediator of podocytopathy in HIVAN), as well as pathways less well described in podocytes. These data provide important insight into the function of NMMHCIIA within podocytes and how NMMHCIIA dysfunction contributes to podocytopathy. Additionally, PPIs we identified have broad implications for the function of actin-myosin complexes within diverse cell types and disease pathways, including the pleiotropic phenotype of MYH9RD.

The non-random overlap of 26 proteins found in both the murine and human screens include many well characterized cytoskeletal proteins. These include proteins known to link the cytoskeleton to the plasma membrane such as integrin, radixin, moesin and talin. This overlap also included a number of myosins, including NMMHCIIA. Two conclusions can be drawn from this overlapping data. First, it demonstrated a non-random reproducibility between our two analyses. Second, the inclusion of well characterized cytoskeletal regulators found in both species samples likely represent proteins with evolutionarily-conserved functions. Specifically, *MYH10* and *MYH14,* which encode NMMHCIIB and NMMHCIIC respectively, were found in the screens in both species. These are well characterized myosin II heavy chains which are known to dimerize with NMMHCIIA in myosin II filaments [2]. Additionally, *MYL12B* was found in both screens. This encodes a regulatory light chain known to function within myosin IIs [2]. The isolation of these gene products within NMMHCIIA complexes from murine and human podocyte lines speaks to their evolutionarily conserved, essential functions in cell contractility.

We believe that further analysis into the functions of these proteins will provide important insights into podocyte function, and the role of NMMHCIIA within the podocyte.

Our screen identified the Rho pathway, which stabilizes the formation of actin-myosin filaments into stress fibers necessary for maintaining podocyte structure and function [48], [49]. Loss of RhoA signaling is a feature of podocytopathy in HIVAN [45], [46]. Excessive RhoA activation in mice leads to the development of FSGS, indicating the necessity for tight regulation of the Rho signaling pathway [50]. Identification of this pathway makes possible further investigation of a feedforward or feedback mechanism between RhoA and NMMHCIIA, which could have important implications for podocyte biology.

We also identified *nef*-regulated proteins as enriched within identified proteins. *Nef*, an accessory gene of HIV-1, mediates podocyte cytoskeletal reorganization during HIVAN [46]. *Nef* contains a Src homology 3 (SH3)-binding domain, which mediates Src kinase activation, and activation of Stat3 and MAPK1,2 pathways [51]–[53]. The role of *nef* in mediating podocytopathy, and previous demonstration that *MYH9* is down-regulated by HIV-1 in the podocyte [11], indicates that *MYH9* down-regulation is likely part of the same *nef*-mediated signaling cascade that includes RhoA down-regulation. The PPIs identified in this analysis could have important therapeutic implications for the treatment of *nef*-mediated pathogenesis in HIVAN, especially given the implications that loss of NMMHCIIA function mediates renal disease.

In order to corroborate our *in vitro* analysis, we utilized immunohistochemistry to analyze the spatial expression of NMMHCIIA in glomeruli. Podocyte filtration function is dependent upon the slit diaphragm apparatus supported by actin cytoskeleton, disruption of which leads to podocytopathy and renal insufficiency [54]. Our demonstration of *in vivo* NMMHCIIA expression within podocyte foot processes supports the hypothesis that dysregulation in NMMHCIIA function leads to podocytopathy.

We analyzed the podocyte cytoskeleton following depletion of *MYH9* expression. Knockdown of *MYH9* expression in podocytes resulted in rarefied actin content, with loss of robust actin stress fibers. Consistently, in previously described siRNA-mediated knockdown of *MYH9* in HEK 293 cells, loss of *MYH9* expression resulted in disruption of the actin cytoskeleton, loss of stress fibers and loss of focal adhesions [55]. The observed effects in podocytes confirm that *MYH9* is necessary for podocyte cytoskeletal organization. Additionally, HIV-1 transgenic podocytes demonstrate a *nef*-dependent, RhoA-mediated loss of podocyte cytoskeletal organization with loss of stress fibers [45]. The finding that loss of *MYH9* expression is sufficient to recapitulate this phenotype, combined with previous demonstration that *MYH9* expression is reduced in the setting of HIVAN [11], supports the hypothesis that loss of NMMHCIIA function is a pathogenic mediator of podocytopathy in HIVAN.

Our study provides the first systematic insight into the protein-level functions of NMMHCIIA-enriched complexes within the podocyte. The podocyte is a unique cell characterized by a dynamically contractile and complex actin cytoskeletal arrangement, and by the expression of many podocyte-specific proteins. Structural dysregulation of the podocyte cytoskeleton and supported proteins is thought to be the primary trigger of a variety of glomerular diseases that affect millions of people including HIVAN, hypertensive nephropathy, diabetic nephropathy, minimal change disease, membranous nephropathy, and similar disorders. Understanding the role of NMMHCIIA in regulating podocyte cytoskeletal dynamics holds great promise toward the development of new generations of therapy.

More broadly, our analysis identified PPIs and functional pathways that may mediate NMMHCIIA’s role beyond the podocyte. While the clinical phenotypes of MYH9RD, including sensorineuronal hearing loss, macrothrombocytopenia, leukocyte inclusion bodies and cataracts are well characterized, the molecular mechanisms underpinning these changes remain largely unknown. Our identification of genes linked to the OMIM disease term deafness may provide functional insight into the phenotype of MYH9RD. In testing hypotheses related to NMMHCIIA’s function within the podocyte, our analysis secondarily generated a broad list of proteins and functional networks which will likely shed light onto NMMHCIIA’s other functions.

## Supporting Information

Table S1 **Murine NMMHCIIA-interacting proteins.**
(XLS)

Table S2 **Human NMMHCIIA-interacting proteins.**
(XLS)

Table S3 **Enrichment analysis of murine pathways represented in NMMHCIIA-interacting proteins.**
(XLS)

Table S4 **Enrichment analysis of human pathways represented in NMMHCIIA-interacting proteins.**
(XLS)

Table S5 **Enrichment analysis of CORUM protein complexes in human community structure analysis.**
(XLS)
